# Safety and Immunogenicity of a Human Epidermal Growth Factor Receptor 1 (HER1)-Based Vaccine in Prostate Castration-Resistant Carcinoma Patients: A Dose-Escalation Phase I Study Trial

**DOI:** 10.3389/fphar.2017.00263

**Published:** 2017-05-10

**Authors:** Iraida Caballero, Lazaro E. Aira, Anabel Lavastida, Xitlally Popa, Javier Rivero, Joaquín González, Mónica Mesa, Narjara González, Kelly Coba, Patricia Lorenzo-Luaces, Barbara Wilkinson, Yuliannis Santiesteban, Yanela Santiesteban, Mayelin Troche, Eduardo Suarez, Tania Crombet, Belinda Sánchez, Angel Casacó, Amparo Macías, Zaima Mazorra

**Affiliations:** ^1^Department of Oncology, Hermanos Ameijeiras HospitalHavana, Cuba; ^2^Department of Clinical Immunology, Center of Molecular ImmunologyHavana, Cuba; ^3^Center for Medical-Surgical ResearchHavana, Cuba; ^4^Tumor Immunology Direction, Center of Molecular ImmunologyHavana, Cuba; ^5^Faculty of Medicine “Victoria de Girón”Havana, Cuba; ^6^Clinical Trials Direction, Center of Molecular ImmunologyHavana, Cuba; ^7^Department of Innovation, Center of Molecular ImmunologyHavana, Cuba

**Keywords:** prostate cancer, immunogenicity, safety, therapeutic vaccine, human epidermal growth factor receptor

## Abstract

Metastatic castration-resistant prostate cancer (CRPC) remains incurable due to the lack of effective therapies. Activation of the human epidermal growth factor receptor 1 (HER1) in prostate cancer contributes to metastatic progression as well as to disease relapse. Here, we determined the toxicity and immunogenicity of a HER1-based cancer vaccine in CRPC patients included in a phase I clinical trial. CRPC patients (*n* = 24) were intramuscularly vaccinated with HER1 vaccine consisting of the extracellular domain of HER1 molecule (ECD) and very small size proteoliposome from *Neisseria meningitidis* (VSSP) and Montanide ISA-51 VG as adjuvants. Patients were included in five groups according to the vaccine dose (100, 200, 400, 600, and 800 μg). The primary endpoints were safety and immunogenicity. The anti-HER1 antibodies were measured by an ELISA, the recognition of an HER1 positive tumor cell line and the inhibition of HER1 phosphorylation by sera were determined by flow cytometry and western blot analysis, respectively. The HER1-specific T cell response was assessed by determination of IFN-γ-producing T cells using ELISpot assay. The vaccine was well tolerated. No grade III or IV adverse events were reported. High titers of anti-HER1 antibodies were observed in most of the evaluated patients. There were no significant differences regarding the geometric means of the anti-HER1 titers among the dose groups except the group of 100 μg in which antibody titers were significantly lower. A Th1-type IgG subclasses pattern was predominant in most patients. Only patients receiving the higher doses of vaccine showed significant tumor cell recognition and HER1 phosphorylation inhibition by hyperimmune sera. Forty two percent of the patients showed a specific T cell response against HER1 peptides pool in post-treatment samples. There was a trend toward survival benefit in those patients showing high anti-HER1 specific antibody titers and a significant association between cellular immune response and clinical outcome.

## Introduction

In developed countries, prostate cancer is the second most frequently diagnosed cancer in men (Ferlay et al., 2015). Its development starts from epithelial cells in the peripheral zone of the prostate, where the cancer develops slowly and remains localized. Then, this organ can be crossed and prostate cancer becomes invasive, leading to metastasis in lymph nodes and later in the bones, liver, and lungs (Bubendorf et al., 2000).

Although prostate cancer is a neoplasia generally sensitive to androgen deprivation (Heidenreich et al., 2011), it has been demonstrated that after a non-fixed period, several patients evolve to a new form of this disease called castration-resistant prostate cancer (CRPC), which leads to an increased mortality. CRPC is not a single, homogenous disorder, but rather a spectrum of clinical states ranging from asymptomatic or minimally symptomatic, non-metastatic disease to symptomatic disease with metastases. Although each patient’s disease course may be different in terms of timing, there is a general progression from asymptomatic to symptomatic disease and potential death (Silvestri et al., 2016).

In 2005, docetaxel in combination with prednisone was approved by the US Food and Drug Administration (FDA) on the basis of an OS benefit, which was demonstrated in 2 phase III studies (Petrylak et al., 2004; Tannock et al., 2004; Berthold et al., 2008). Subsequently, docetaxel-based chemotherapy became the standard of care for patients with mCRPC (metastatic CRPC), although its use was limited because the toxicity profile especially in older age patients. The established role of docetaxel in the treatment of mCRPC created three possible moments for new treatment options: pre-docetaxel, in combination with docetaxel, and post-docetaxel. Many treatment options in combination with docetaxel have failed to show any benefit. Especially asymptomatic or minimally symptomatic mCRPC patients are considered eligible for pre-docetaxel treatment to postpone the initiation of cytotoxic chemotherapy (Cornford et al., 2017).

Despite the improved therapeutic options for CRPC, new treatments are still needed to grant durable disease control and long-term survival benefit with minimal toxicity. Among the many strategies that are being investigated to address this issue, immunotherapy is a compelling approach. Research suggests that prostate cancer is an immunologically modulated malignancy, and therefore, may be sensitive to immunotherapy. For example, data from studies that have evaluated the cellular composition of prostate tumors suggest that immune cell populations infiltrate the prostate gland (Gannon et al., 2009; May et al., 2011). Infiltrating leukocytes detected in prostate tumors include natural killer cells, effector cells, and regulatory T cells, suggesting that both the innate and adaptive branches of the immune system may play a role in mounting an attack against prostate cancer cells (Cha and Small, 2013).

Immunotherapy for prostate cancer uses a wide variety of approaches such as therapeutic vaccines and anti-checkpoints inhibitors. Monoclonal antibodies directed to the immune checkpoints molecules CTLA-4 and PD-1 are being used in CRPC patients. In a phase III clinical trial for mCRPC patients the addition of the anti-CTLA-4 MAb Ipilimumab (Yervoy; Bristol-Myers Squibb, New York, NY, USA), to radiotherapy did not show any improvement in OS (Kwon et al., 2014). Currently, combination trials using ipilimumab are underway. In addition, the anti-PD1 MAbs, Nivolumab (Opdivo; Bristol-Myers Squibb, New York, NY, USA) and Pembrolizumab (Keytruda, Merck Sharp, and Dohme Corp) are under evaluation in phase I clinical trials.

The four main types of vaccine-based immunotherapies studied in CRPC can be classified as autologous, cell based, viral vector based, and DNA vaccines (Gerritsen and Sharma, 2012). Most of them are being used for asymptomatic or minimally symptomatic CRPC patients (Noguchi et al., 2016). Autologous vaccines, such as Sipuleucel-T (Provenge, Dendreon Corp, Seattle, WA, USA) which targets prostatic acid phosphatase has demonstrated a considerable specific T-cells activation and a reduction in the PSA (Burch et al., 2000). Moreover, the recombinant viral vaccine, PROSTVAC-VF/TRICOM, which targets the PSA, is currently under evaluation in different clinical trials. In a randomized phase II clinical trial comparing men receiving the vaccine with men who received placebo, a survival advantage for the vaccine group was obtained (Kantoff et al., 2010). Up to now, Sipuleucel-T is the only vaccine approved by FDA on April 29, 2010, to treat asymptomatic or minimally symptomatic mCRPC. Hence, therapeutic cancer vaccines have arisen as a new strategy to induce antitumor response.

Human epidermal growth factor receptor (HER1) is ubiquitously expressed in the human body. This molecule is recognized as a tumor-associated antigen because it is overexpressed in many kinds of human cancers including prostate tumors (Speake et al., 2005). Indeed, a growing body of evidences indicate that activation of the HER1 in prostate cancer contributes to metastatic progression as well as to disease relapse (Hernes et al., 2004). Immunohistochemical analyses have shown an increase in HER1 expression during prostate cancer development. In addition, a correlation with tumor recurrence and advanced stage disease has been established (Ko et al., 2003). Several investigators demonstrated HER1 expression as high as 90–100% in tissue from patients with mCRPC (Scher et al., 1995; Di Lorenzo et al., 2002). These findings suggest that EGFR-targeted drugs could be of therapeutic relevance in the management of advanced prostate cancer.

Despite the high expression of HER1 in CRPC, the inhibition of this pathway has not been perceived as a valid target in the treatment of CRPC. Most previous clinical trials with HER1–tyrosine kinase inhibitors or monoclonal antibodies did not show any significant activity in the referred setting. Different reasons could provoke the lack of effectiveness. Clinical relevance toxicities using tyrosine kinase inhibitors have conducted to interruption or doses reduction (Pezaro et al., 2009). Besides, predictive biomarkers must be identified to select the clinically benefited population. In this sense, Cathomas et al. (2012) reported significantly improved efficacy for the HER1 monoclonal antibody cetuximab in patients with overexpression of the receptor and persistent expression of PTEN.

Taking into account this knowledge, we developed a therapeutic cancer vaccine based on the extracellular domain (ECD) of the HER1, which was adjuvanted in VSSP and Montanide ISA 51 VG. The rational of this vaccine-based immunotherapy was to stimulate an anti-EGFR immune response against EGFR- expressing tumor cells with minimally collateral damage to normal tissues. Previous non-clinical studies demonstrated that immunization of mice with HER1-ECD combined with VSSP induced highly specific IgG antibodies with strong *in vitro* cytotoxic effect over HER1 human cell lines. In addition, self-immunization of mice using murine EGFR-ECD promoted not only a highly specific immune response but also a potent anti-metastatic effect in the EGFR+ Lewis lung carcinoma model (Ramirez et al., 2008). Regarding to vaccine safety, immune response induced in vaccinated mice did not have a deleterious effect in wound healing process (Fuentes et al., 2014) and reproduction-associated side effect was absent (Ramirez et al., 2006). Besides, toxicity studies in rats and monkeys demonstrated that vaccine was immunogenic and well tolerated with only local reactions at administration site (Barro et al., 2012; Mancebo et al., 2012). Based on these findings, a first -in- human phase I clinical trial was approved in 2009 by the Cuban Regulatory Agency (CECMED).

Here, we show the results of a single arm, dose escalation, open-label, phase I clinical trial in asymptomatic or minimally symptomatic CRPC patients. The main endpoints were safety, dose-limiting toxicities and immunogenicity of the HER1-based therapeutic cancer vaccine. The preliminary association between immunological parameters and clinical benefit was also evaluated.

## Patients and Methods

### Test Substance

HER1-ECD (human epidermal growth factor receptor-ECD) vaccine was release by the Quality Control Department from the Center of Molecular Immunology in Havana, Cuba. The vaccine consisted of 100, 200, 400, 600, and 800 μg of HER1 adjuvanted in 100 μg of VSSP and emulsified on Montanide ISA-51 VG in a proportion 1:1 (v/v) immediately before injection.

### Patients’ Selection

Eligible patients were 40 years or older with CRPC histologically confirmed. All patients had an Eastern Cooperative Oncology Group performance status (ECOG PS) ≤ 2 with a life expectance of at least 6 months, as well as adequate renal, hepatic, and hematologic functions. Exclusion criteria included patients who received any prior chemotherapy, patients with uncontrolled chronic diseases or with active infections, patients with positive serology for hepatitis B, C, and for HIV, and patients with central nervous system metastases.

### Study Design

The uncontrolled, dose escalation, open-label, phase I clinical trial was approved by the ethic review boards from the Center for Medical-Surgical Research and from the Hermanos Ameijeiras Hospital, both hospitals in Havana, Cuba. The study protocol was conducted in accordance to the principles of the Declaration of Helsinki and Good Clinical Practices guidelines and under the Investigational New Drug application authorized by the Cuban Regulatory Agency (CECMED). All patients provided written informed consent.

The study consisted in a dose escalation protocol with five level dose groups (100, 200, 400, 600, and 800 μg) of HER1 vaccine. Patients were vaccinated by the intramuscularly route and received nine doses of the HER1 vaccine for a period of 6 months. The induction phase consisted of five doses administered every 2 weeks and then, patients were vaccinated every 4 weeks. According to the protocol, if at any time within 28 days of vaccination, two patients or more developed severe related adverse events, the previous dose level was considered the maximum tolerated dose. Other concomitant antitumor therapies were not permitted.

All patients included in the study who received at least three doses of the HER1 vaccine were selected for immunological response evaluation, provided that they had the pre-immune and at least two post-immune samples. PBMC and serum samples were taken from patients before receiving each vaccination and every 28 days after completing the administration regimen up to 1 year follow up.

### Safety and Tolerability

All patients included in the study were evaluated for safety. The frequency, nature, causality, and severity of the adverse events were evaluated at each dose level. Severity was graded according to the NIH Common Terminology Criteria for Adverse Events, version 3.0. Special attention was given to administration related symptoms and allergic reactions. Laboratory assessments including PSA level were performed during the 6 months of administration period and up to 1 year.

### Measurement of Antibody Response

A sandwich ELISA determined the antibody response against HER1-ECD. Ninety-six-well microtiter plates (Corning Inc.) were coated with 5 μg/ml of HER1-ECD and then blocked with PBS-Tween 0, 05% containing 1% BSA. Sera were added at 1:100 dilution and then serial 1:2 dilutions were performed. After, alkaline phosphatase-conjugated anti-human IgG (γ-chain specific) antibody (Sigma) was added. Reaction was developed with the p-nitrophenylphosphate substrate (Sigma) in diethanolaminebuffer, pH 9.8, stopped with 3 M NaOH and read at 405 nm in a microplate reader (Thermo Scientific). The inverse of the highest serum dilution giving optical density (OD) values > 0, 25 and twice the value of the pre-immune serum was considered as the antibody titer. Assay was performed in triplicates for each sample and the anti-HER1 MAb nimotuzumab at 10 μg/ml was used as assay control.

For measurement of IgG subclass-specific to HER1-ECD, IgG subclass-specific mouse anti-human IgG1, IgG2, IgG3, and IgG4 monoclonal antibodies (IgG1: 9052-05, IgG2: 9070-05, IgG3: 9210-05, and IgG4: 9190-05; Southern Biotech, Cambridge, UK) were used as secondary antibodies, and HRP-conjugated goat anti-mouse IgG antibody (#W4028, Promega, Madison, WI, USA) was used as the third antibody.

### Antibody-Binding Assay by Flow Cytometry

A431 cells were blocked in PBS containing 1% BSA for 30 min on ice. Patients’ serum samples, corresponding to pre-immune (PI) and hyperimmune sera from the sixth, seventh and the last immunization (ninth), as well as sera from the following time-point days after ending treatment, were diluted 1:10 and incubated with 10^5^ cells for 30 min on ice. After washing, cells were incubated with FITC-conjugated goat anti-human IgG antibodies (Jackson Immuno Research Laboratories) diluted 1:100 for 30 min on ice. Percentage of positive stained cells was determined in a FACScan flow cytometer (Becton Dickinson). The FlowJo program (version 5.7.2) was used to analyze the cells acquired on every FACS assay. Positive patients had a binding percent > 20%, after subtracting the pre-immune to the recognition of hyperimmune sera. Nimotuzumab at 10 μg/ml was used as positive assay control.

### HER1 Phosphorylation-Inhibition by Patient Sera

An immunoblotting assay, which detects phosphorylated HER1, was used to evaluate the capacity of the anti-HER1-ECD antibodies to inhibit the HER1 activation in the presence of epidermal growth factor (EGF). A431 human epidermoid carcinoma cell line (American Type Culture Collection) were serum starved for 12 h and then incubated with sera diluted 1/100 at different time points from vaccinated patients for 1 h at 37°C. Tyrosine kinase inhibitor (Tyrphostin AG1478) at 1 mol/L was used as positive control. Cell lysates were prepared using 50 mmol/L HEPES (pH 7.4), 0.15 mol/L NaCl, 1% Triton X-100 buffer containing 1 mmol/L EDTA, 1 mmol/L EGTA, 1 mmol/L phenyl- methylsulfonyl fluoride, and 1 mmol/L Na_3_VO_4_, and then clarified by centrifugation. The protein concentration of the lysates was determined with a bicinchoninic acid protein assay kit (Pierce). Equal amounts of protein were resolved on SDS-PAGE, transferred onto polyvinylidene difluoride nitrocellulose membrane (Gelmar), followed by blocking with NEGT buffer [0.15 mol/L NaCl, 5 mmol/L EDTA, 50 mmol/L Tris- HCl (pH 7.5), 0.02% Tween 20, and 0.04% gelatin] overnight at 4°C. Then the membranes were incubated with specific anti-phosphotyrosine antibody (Santa Cruz Biotechnology) at room temperature for 1 h. After washing with NEGT buffer, the membranes were incubated with secondary antibody (anti-mouse or anti-rabbit antibodies conjugated with horseradish peroxidase) for 1 h at room temperature. The signal was visualized by enhanced chemiluminescence according to the manufacturer’s instruction (Amersham Biosciences, UK) and band intensity was quantitated using a personal densitometer SI (Pharmacia Biotech) and ImageMaster 1D prime Software. To normalize the protein loading on the gel, the membranes were stripped and reprobed with anti-EGFR antibodies for 1 h at room temperature. Anti-mouse antibodies conjugated with horseradish peroxidase were used as secondary antibodies. ECL plus Western blotting detection reagents (Amersham Biosciences) were used as detection system. The inhibition of phosphorylation occurred when values were higher than the mean of the percentages of inhibition reached with the pre-immune sera plus 2 SD.

### Enzyme-Linked Immunosorbent Spot (ELISpot) Assay

IFN-γ-secreting PBMCs were detected using an ELISpot kit (Mabtech AB). PBMC seeded at 2^∗^10^6^ cells/ml in a U-bottom plate (Greiner Bio-One) were stimulated at 10 μg/ml from a pool of 14 peptides [(1) ITDFGLAKL; (2) KLFGTSGQK; (3) YLNTVQPTC; (4) TSLGLRSLK; (5) KTIQEVAGY; (6) KVCQGTSNK; (7) MFNNCEVVL; (8) MYYENSYAL; (9) KEITGFLLI; (10) TPPLDPQEL; (11) FLKTIQEVA; (12) VQRNYDDLSF; (13) QFSLAVVSL; and (14) ENNTLVWKY]. Peptides were determined in Base Synthetic Software taking into account those with higher binding for HLA class I, and all peptides were synthesized by the Center of Genetic Engineering and Biotechnology, Havana, Cuba. Then, cells were re-stimulated every 3 days with the same pool of peptides and human recombinant IL-2 (ebiosciences, Birmingham, UK) (25 UI/ml) until 10 days of stimulation. Afterward, the well contents were transferred to a pre-coated IFN-γ ELISpot plate and incubated for 24 h at 37°C. Then, the plate was washed six times with filtered PBS. Binding was revealed by adding a biotinylated anti-IFN-γ antibody followed by alkaline phosphatase-conjugated streptavidin. After adding the substrate and stopping the reaction with running water, the plate was dried at room temperature and the spots were quantified using the ImmunoSpot Analyzer software (AID-ELISpot 5.0 software, AID). The number of spot-forming units (SFU) per 2^∗^10^5^ PBMC was calculated by subtracting non-specific values (spots in wells with unstimulated cells). Response definition was set arbitrarily taking into account the self-nature of the HER1. A response of 50 spots or less was considered low response, between 51 and 99 SFU was considered medium response and a response of 100 spots or more was considered a high response (Janetzki et al., 2008).

### Statistical Analyses

Kolmogorov–Smirnov test was used to explore normality of data. Generalized estimating equation was used to compare titer curves between groups. Bonferroni correction was used in multiple comparisons. Quasi-Likelihood-Based under the independence model criterion was used for model selection (Pan, 2001). The means of post-immune sera recognition after baseline values subtraction were calculated for each patient. Mann–Whitney test was used to compare Inhibition of HER1 phosphorylation by sera at post-immune time point vs. the baseline. Survival data were analyzed using the Kaplan–Meier method and the log-rank test was applied to explore the differences in OS associated to immunological variables. Statistical analyses were made with SSPS program (version 16.0). The signification level was assumed as 0.05 for all the hypothesis tests.

## Results

### Patients’ Characteristics

From October 23, 2009 to August 2015, 24 patients were included in this study. Three patients were included in the group of 100 μg and then, the dose was scaled up to the next level (200 μg) since no adverse events were detected in the first cohort. Five patients were included in the group of 200 μg, six in the 400 μg, five in the 600 μg, and five in the 800 μg. Demographic and baseline characteristics were comparable among groups (**Table 1**).

**Table 1:** Demographic and baseline characteristics.

Groups	100 μg	200 μg	400 μg	600 μg	800 μg	*n* (%)
No. Patients	3	5	6	5	5	
Age Mean years (SD)	72.5 (7.7)	63.2 (6)	67.3 (8.4)	64.8 (5.9)	66.3 (8.2)	
Race, No						
Caucasian	2	0	3	1	3	37.5
African–American	0	1	0	3	0	16.7
Mulatto	1	4	4	1	1	45.8
ECOG, No. (%)						
0	1	5	5	1	2	58.3
1	2	0	1	4	3	41.7
Prior, therapy						
Surgery	1	0	1	2	1	20.8
Chemotherapy	0	0	0	0	0	0
Radiotherapy	0	4	3	4	2	54.2
Hormonotherapy	2	5	5	3	4	79.2
Serum PSA level, (ng/ml)						
Mean (*SD*)	54,2 (29,6)	221,9 (276.6)	21,1 (18.2)	98 (4.5)	31,8^∗^	
Median (range)	60,8 (22–80)	88,6 (87–716)	15,3 (8–57)	100 (90–100)	22 (3–79)	

*PSA, prostate-specific antigen. ^∗^Baseline PSA was missing in one patient from group 5 (*n* = 4).*

All patients received nine immunizations of HER1 vaccine. Six patients followed for less than 1-year due to different causes: four patients had symptomatic progression and were taken off the study; one subject died after progressive disease and the last patient voluntarily abandoned the trial.

### Safety

No patient experienced dose-limiting toxicity, therefore the maximum tolerated dose was not reached. There were no deaths attributed to vaccination. A total of 148 adverse events were reported: 47 in the group of 200 μg, 35 in the 400 μg, 45 in the 600 μg, and 21 in the group of 800 μg. No adverse events were reported in the group of 100 μg. No correlation was found between the adverse events and the vaccine dose. Only 16 treatment-related adverse events were reported in 21% of patients. The most common adverse events were injection site reactions, fever and those attributed to the natural course disease such as bone pain, anemia, increase in phosphatase alkaline and asthenia. No severe adverse events related to treatment were described.

### Humoral Immune Response Induced by HER1 Vaccine

**FIGURE 1:**
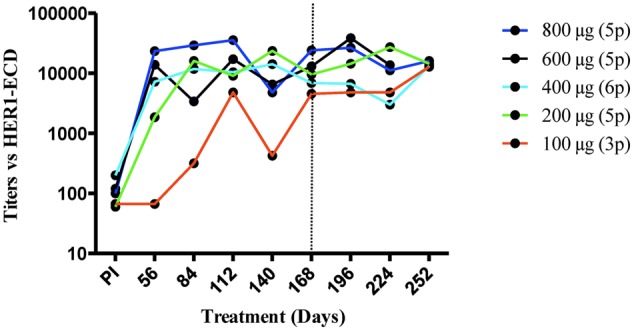
**Anti-HER1-ECD antibody response.** Antibody titers were determined by an ELISA in serially diluted sera from patients vaccinated with different doses of HER1vaccine. Each line with different colors represents the geometric mean of anti-HER1-ECD antibody titers for patients belong to each dose at different time points. Perpendicular line shows the end of vaccination period. Regarding to antibody titers not significant difference where found among the groups but 100 μg, in which titers were significantly lower (vs. 200 μg, *p* = 0,011; vs. 400 μg, *p* = 0,045; vs. 600 μg, *p* = 0,029 and vs. 800 μg, *p* = 0,023; generalized estimating equation).

Antibody response against HER1-ECD was repeatedly measured in all patients for every dose. Most vaccinated patients (22 out of 24) developed anti-HER1-ECD specific IgG antibody response (mean for all doses: 1:14,500). Antibody titers in the group immunized with the lower dose of vaccine (100 μg of HER1-ECD) was significantly lower as compared with the other doses (vs. 200 μg, *p* = 0,011; vs. 400 μg, *p* = 0,045; vs. 600 μg, *p* = 0,029 and vs. 800 μg, *p* = 0,023; generalized estimating equation). The antibody titers induced in patients vaccinated with different doses of vaccine (200, 400, 600, and 800 μg) were very similar (*p* > 0,05; generalized estimating equation). Patients who received higher doses of HER1 vaccine (400, 600, and 800 μg) showed a more rapid onset of the antibody response (2 months). In all groups but 100 μg of HER1-ECD, the antibody response reached a plateau between 2 and 3 months after starting immunizations. Even though the patients were vaccinated during 6 months, the immunoglobulin response remained high 3 months after ending the immunization period in most patients. **Figure 1** depicted the geometric mean of antibody response to HER1-ECD in each group.

**FIGURE 2:**
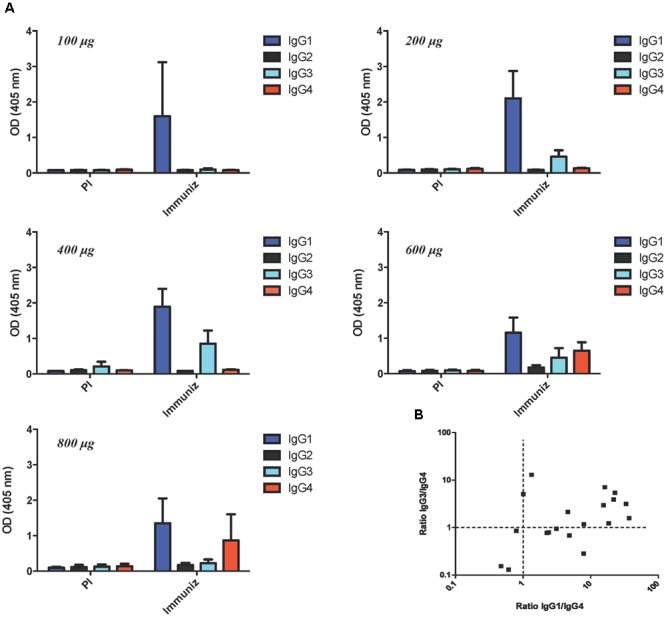
**Distribution of anti-HER1-ECD IgG antibody subclasses. (A)** Evaluation of IgG subclasses at baseline and day 112 in each group of patients vaccinated with different doses of HER1 vaccine. Serum levels of anti-HER1-ECD IgG1, IgG2, IgG3, and IgG4 antibodies were measured by ELISA using subclasses-specific secondary antibodies. **(B)** Two dimensional plot of the ratio Th1 to Th2 anti-HER1-ECD IgG subclasses. Ratio of absorbance of IgG1 or IgG3 antibody regarding to IgG4 antibody for each patient sample was plotted. The majority of patients developed a Th1-type subclass pattern.

To study whether the HER1-specific immune response was Th1-type, dominant subclasses of specific IgG antibodies were analyzed in 20 out 22 patients. These subjects had developed IgG response after vaccination and had available samples. Baseline and hyperimmune sera obtained at day 112 (around 4 months) after starting vaccination were evaluated. Most patients showed IgG1 as predominant subclass. Interesting, IgG3 subclass was also induced in patients from 400 to 600 μg. In the case of patients receiving 600 and 800 μg, a pattern of IgG4 and IgG1 was induced. Since IgG1 and IgG3 are known as Th1-type IgG subclasses, whereas IgG4 is considered as a Th2-type IgG subclass, the ratio IgG1/IgG4 or IgG3/IgG4 of ≥1 was estimated as indicative of Th1 response. Seventeen out of 20 cases (85%) exhibited Th1-type HER1-ECD specific responses, whereas three patients (2 out of 4 vaccinated with 800 μg and 1 out of 5 with 600 μg) seemed to develop a Th2 pattern after 3–4 months of vaccination. **Figure 2** shows the distribution of specific IgG subclasses at day 112 according to HER1-ECD doses. In patients from 100 to 600 μg, the distribution of subclasses was also evaluated at day 168 due to availability of sera. In this subjects the IgG subclasses profile was very similar as compared to day 112 (data not shown).

### Functionality of the Anti-HER1-ECD Induced Antibodies

**FIGURE 3:**
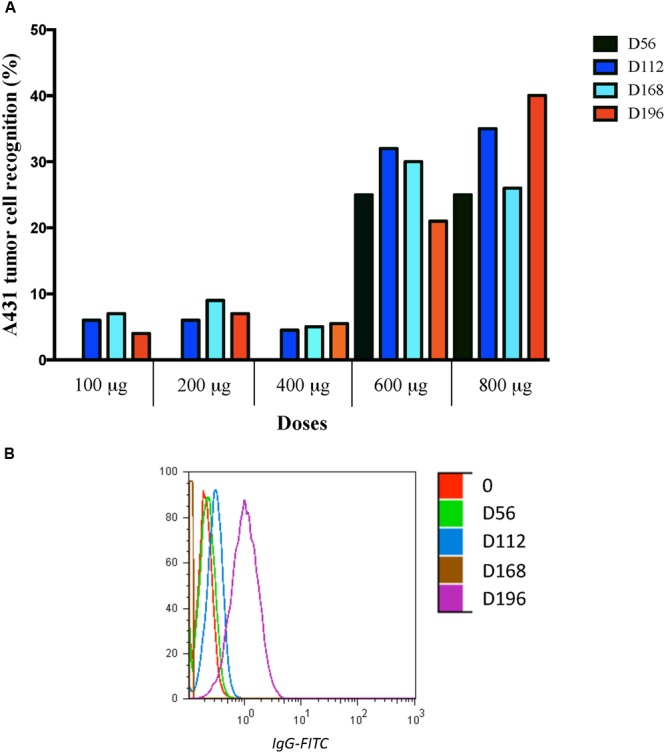
**Binding of pre-immune and hyperimmune sera of vaccinated patients to the A431 cells.** Recognition assay was performed by incubating the 1:10 diluted sera with A431 cells at different time points, in the pre-immune (PI), in days 56, 112, and 168, as well as 28 days after ending treatment (Day 196). **(A)** Mean of post-immune recognition after baseline values subtraction obtained for all patients in every dose. Recognition of A431 cells (>20%) was seen in patients received higher doses of vaccine. **(B)** Histogram of A431 recognition by sera from one representative patient belonging to 800 μg is shown.

To assess the ability of induced antibodies to recognize the HER1 in the natural context of tumor cell membranes, patients’ sera binding to HER1-overexpressed A431 cells, was tested. Pre and hyperimmune sera from all vaccinated patients were evaluated. Notably, hyperimmune sera from patients vaccinated with 100, 200, or 400 μg of HER1-ECD did not show significant recognition as compared with baseline values (<20% recognition of tumor cells). Post-immune sera from patients vaccinated with higher doses (600 and 800 μg) showed a modest recognition of HER1 positive cell line (25–30% recognition of tumor cells) (**Figure 3A**). Anti-HER1 MAb nimotuzumab, which highly recognizes A431 cells, was used as positive control (data not shown). The recognition of A431 tumor cell line by the sera from a representative patient (800 μg group) is shown in **Figure 3B**.

**FIGURE 4:**
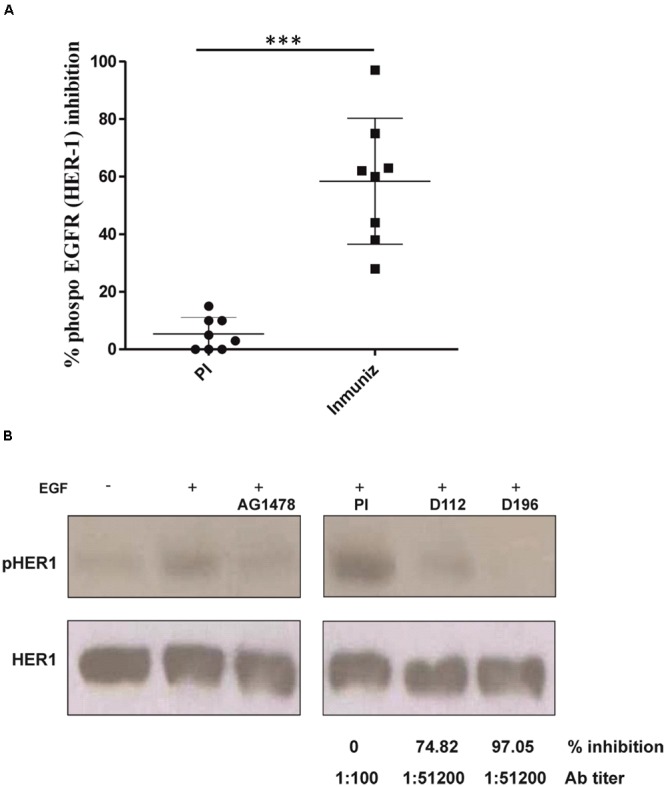
**Inhibition of HER1phosphorylation in A431 tumor cell line by sera from immunized patients. (A)** The assay is only presented with eight positive patients’ sera from doses of 400, 600, and 800 μg. HER1 phosphorylation inhibitor AG1478 (10 μM) was used as positive control and A431 cells were stimulated with human recombinant EGF (100 ng/ml). Mean of inhibition percentages of HER1 phosphorylation at pre-immune sera and after immunization (3–4 months) is shown. The relation between the densitometry units obtained with the anti-phosphotyrosine antibody and the same blot reproved with anti-HER1 antibody was used to set the real activation status. Inhibition is defined as the fraction of HER1 phosphorylation at the given time point compared with the HER1 phosphorylation with the addition of EGF and without treatment with patient’s sera and it was considered 100% of HER1 activation. Inhibition of HER1 phosphorylation at post-immune time point was significantly higher as compared to baseline (*p* ≤ 0.05, Mann–Whitney *U* test). **(B)** Representative immunoblots showing the phosphorylation levels of HER1 in cells incubated with sera from a vaccinated patient (600 μg) at different time points. ^∗∗∗^*p* < 0.05.

The capacity of anti-HER1-ECD antibodies to inhibit the activation of HER1 in the presence of EGF was examined by Western blotting assay. No significant inhibition of HER1 phosphorylation was achieved in patients immunized with 100 or 200 μg. Hyperimmune sera from 8 out 11 evaluated patients (72%) significantly reduced HER1 phosphorylation in the presence of EGF (range, 28.1–97.06% of inhibition) as compared with the pre-immune serum (*p* < 0,001) (**Figure 4A**). Results are shown only for patients receiving doses of 400, 600, and 800 μg of HER1 vaccine. A western blot from a representative patient vaccinated with 600 μg of HER1-ECD is shown in **Figure 4B**. In this case, a complete abrogation of the HER1 phosphorylation was obtained (anti-HER1-ECD titer: 1:51200) at the end of immunization period (6.5 months).

### Cellular Response Induced by HER1 Vaccine

**FIGURE 5:**
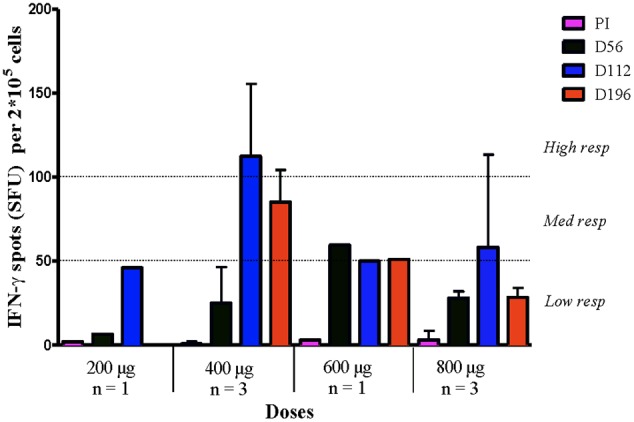
**T cell response to HER1-ECD peptides in patients vaccinated with HER1 vaccine at different doses.** PBMC were stimulated with a pool of peptides (see Patients and Methods) from HER1-ECD and IFN-γ-producing T cells in response to stimulation was tested by ELISpot. Numbers of spots-forming units (SFU) were counted at pre-immune (PI), day 56, day 112 and after 28 days of ending treatment (Day 196). Means ± SD are represented. Responses were considered positive if 10 or more specific spots were detected and if the number of spots in the presence of an antigen was at least twofold that in its absence. Responses were arbitrarily classified as a low response if the spots were in the range 0–50 spots, medium response if the spots were between 51 and 99 spots and a high response above 100 spots. The number of SFU belonging to responder patients from 200, 400, 600, and 800 μg are shown. IFNγ-T cell response was considered positive in 8 out 19 vaccinated patients.

Available PBMC from 19 patients vaccinated with different doses of HER1 vaccine were stimulated with a pool of peptides (See Patients and Methods). The number of IFN-γ spot forming units (SFU) was evaluated by an ELISpot assay. IFN-γ-T cell response was considered positive in 42% of patients (8 out 19). According to the number of SFU, T cell response was arbitrarily classified in low, medium, and high (Janetzki et al., 2008). Non-significant IFN-γ SFU were observed in the pre-immune sera for all patients. Thus, the responses achieved were a direct consequence of the treatment. For subjects vaccinated with 100 μg of HER1 vaccine no response was found while patients receiving 200 μg only a low response (mean of all patients: 46 SFU) was achieved during the treatment. This response was not maintained after ending vaccination. For patients receiving the doses of 600 and 800 μg, low and medium responses were induced during the treatment (mean of all patients: 40 and 58 SFU; respectively), which was held 1 month after ending immunization (mean of all patients: 31 and 28 SFU; respectively). It is worth to note that the highest response and high frequency of responders (3 out 5, 60%) were found with 400 μg of HER1-ECD after seven immunizations (mean of all patients: 131 SFU). Moreover, 1 month after ending treatment, patients vaccinated with 400 μg of HER1-ECD maintained IFN-γ-secreting T cell response classified as medium response (mean of all patients: 75 SFU) (**Figure 5**). **Table 2** evidences the frequency of patients who showed IFN-γ-secreting T cells corresponding to each HER1-ECD administered dose.

**Table 2:** Number of patients who showed IFN-γ-secreting T cells for each dose.

Doses	Tested patients	Positive (%)	Max response
100 μg	3/3	0/3 (0)	Negative
200 μg	4/5	1/4 (25)	Low
400 μg	5/6	3/5 (60)	High
600 μg	3/5	1/3 (33)	Medium
800 μg	4/5	3/4 (75)	Low–medium

### Immune Response and Relation with Clinical Outcome

**FIGURE 6:**
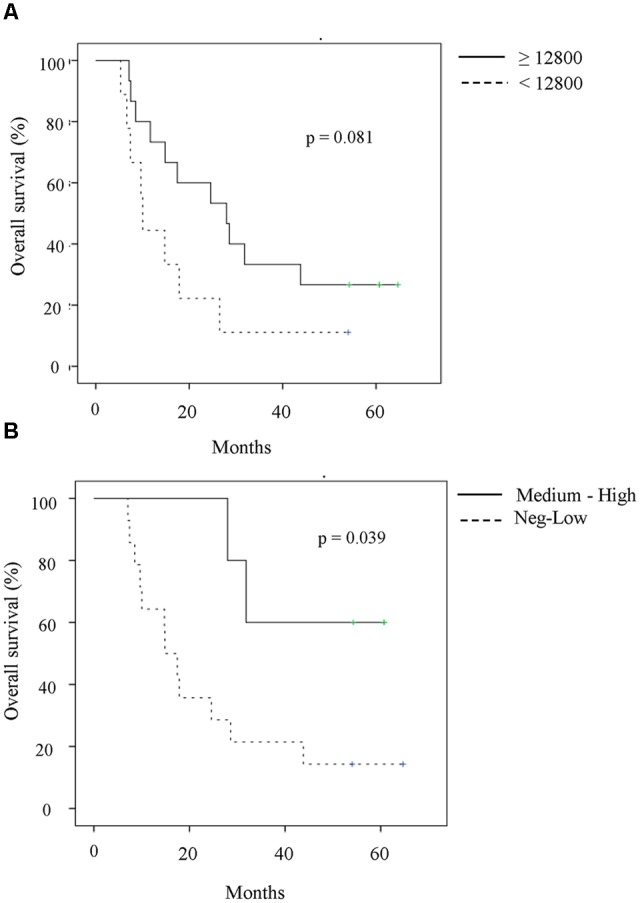
**Association of immune response with clinical outcome. (A)** Survival of patients according to median (12,800) of anti-HER1-ECD antibody titers developed in all the evaluated patients. Patients were divided according to maximum titer reached during vaccination. Although non-significant, patients with antibody titers higher or equal than 12,800 seem to have better survival times as compared to patients with lower antibody response (median, 27.95 vs. 10.02, *p* = 0.081, log rank test). **(B)** Survival of patients according to IFN-γ-secreting T cells measured as SFU. Patients with at least medium number of spot (Dpost-D0 ≥ 50 spots) seem to have better survival in comparison with patients with either lower number of spot (Dpost-D0 < 50 spots) or negative T cell response (Dpost-D0 < 10 spots) (median, not reached vs. 14.85 months, *p* = 0.039, log rank test).

Even though the number of patients was small, the immune response elicited by the vaccine might be related with clinical benefit. Patients who elicited antibody titers higher or equal than 12,800 (median) had a trend toward better survival as compared to patients with lower antibody response (<12,800) (**Figure 6A**). The difference in the median survival was not significant (MST 27.95 vs. 10.02, *p* = 0.081, log rank test), presumably because the small number of patients in each group.

In addition, patients with larger SFU (Dpost-D0 ≥ 50 spots) had significantly better survival in comparison with patients with either reduced number of spots or negative T cell response (Dpost-D0 < 10 spots) (median, not reached vs. 14.85 months, *p* = 0.039, log rank test) (**Figure 6B**). Both results should be confirmed in a larger patient’s sample.

## Discussion

Prostate cancer is the fifth leading cause of deaths from cancer in men (Silvestri et al., 2016). The initial treatment of choice for metastatic prostate cancer is medical or surgical castration. However, metastatic prostate cancer generally acquires resistance to androgen deprivation therapy. According to the American Urological Association guidelines, docetaxel is still the preferred first-line treatment for patients with mCRPC, especially for those with symptomatic mCRPC who have good functional performance and no previous exposure to docetaxel chemotherapy (Gillessen et al., 2015). However, docetaxel can cause severe and potentially life-threatening adverse events compared with hormonal and immune-based therapies. Of these adverse events, neutropenia is one of the major toxicities of docetaxel-based chemotherapy for CRPC patients. It might result in severe infection and therefore require a reduction in the dose of docetaxel, which can potentially compromise the effectiveness of the cancer treatment (Hirasawa et al., 2017).

Other therapeutic options have expanded rapidly. These new agents include drugs that target the androgen axis (enzalutamide, abiraterone), bone seeking radionuclides (radium-223), and second line chemotherapy (cabazitaxel). Based on their efficacy, the newer oral agents that affect the androgen axis have been approved in the pre- and post- docetaxel setting. However, several patients have shown primary resistance to these agents, although the mechanisms of resistance are not fully understood (Buttigliero et al., 2015).

Therefore, additional treatment strategies are needed to further improve the survival outcomes of patients with advanced and metastatic prostate cancer. The manipulation of the immune system represents an attractive alternative to control this tumor (Drake, 2010; Silvestri et al., 2016). In this case, therapeutic cancer vaccines have emerged as a promising strategy to induce an antitumor immune response to shrink tumor and to protect against tumor recurrence or metastatic disease. Different types of vaccines have been used in mCRPC patients. Sipuleucel-T (Provenge) was the first autologous cellular immunotherapy approved by the FDA in 2010 and by the European Medicines Agency (EMA) for the treatment of asymptomatic or minimally symptomatic mCRPC (Sims, 2012), and to date it remains the only FDA-approved immunotherapy for prostate cancer. This vaccine consisted in the use of autologous dendritic cells pulsed with prostate acid phosphatase (Burch et al., 2004) and infused into prostate cancer patients. It induces antigen-specific immune response as well as promotes the recruitment of activated effector T cells in the prostate tumor microenvironment (Agarwal et al., 2012; Wesley et al., 2012). Another promising vaccine is a PSA-targeted poxvirus-based vaccine PROSTVAC. It consists of a heterologous prime-boost (vaccinia or fowlpox virus vector) and three costimulatory molecules (TRICOM; B7.1, ICAM-1, and LFA-3) that serve to increase the PSA-specific immune response (Madan et al., 2009). PROSTVAC-VF/TRICOM was evaluated in a randomized phase II clinical trial in minimally symptomatic mCPRC. The study showed positive results in the median OS with a difference of 8 months between the treatment group (Kantoff et al., 2010). Additionally, an increase in T cell response, greater than sixfold, and a lower Tregs frequency was observed in patients who survived longer.

Here, we conducted a phase I dose-escalation clinical trial in asymptomatic or minimally symptomatic CRPC patients, with a therapeutic cancer vaccine based on the ECD of HER1. The rational of this immunotherapeutic agent was to induce humoral and cellular immune response specific to HER1 molecule, able to recognize and ultimately eliminate HER1 overexpressing tumor cells. Chemotherapy- naïve mCRPC patients were chosen for this first clinical trial. As it has been done in most promising cancer vaccines trials, the selection of this setting is intended to postpone the use of cytotoxic therapy, which is very toxic. Although conflicting data have been reported, some combinations of docetaxel with immunotherapy have failed to prolong OS. A phase II study using ipilimumab vs. ipilimumab and docetaxel did not demonstrate radiologic response (Cha and Small, 2013). Another trial, which administered GVAX vaccine with docetaxel, was stopped prematurely because of lack of benefit (Galsky and Vogelzang, 2010). Since it is a first time using this vaccine in human, the primary endpoints of the clinical trial were safety and immunogenicity.

In cancer patients, high levels of HER1 expression have been associated with fast disease progression and poor survival (Oliveira et al., 2006). Therefore, anti-HER1 therapies with monoclonal antibodies (Pryor et al., 2009; Chen et al., 2013) or tyrosine kinase inhibitors (Dassonville et al., 2007) have been clinically validated for a variety of epithelial tumors demonstrating the capability to inhibit the growth of cancer cells. Previous reports demonstrated very high EGFR expression in tissues from patients with metastatic CRPC (Scher et al., 1995; Di Lorenzo et al., 2002). But up to now, the inhibition of the EGFR family signaling has not been extensively perceived to be a valid target in the treatment of CRPC, despite the high expression of these receptors in advanced CRPC patients. On one hand, clinical relevance toxicities using tyrosine kinase inhibitors have conducted to interruption or doses reduction (Pezaro et al., 2009). On the other hand, since prostate tumors are very heterogeneous, predictive biomarkers must be identified to select the clinically benefited population. In this sense, an exploratory clinical trial (Cathomas et al., 2012) performed in a population of patients pretreated with docetaxel-resistant mCRPC reported significantly improved efficacy in thirty-eight patients with overexpression of HER1 and persistent expression of PTEN.

In our study, we assessed a new therapeutic cancer vaccine to treat mCRPC patients, which is based on the ECD of HER1 combined with two adjuvants: VSSP (from *Neisseria meningitidis*) and Montanide ISA 51 VG. The hypothesis behind the use of HER1-based vaccine is to harness the immune system against EGFR expressing tumor cells with minimal toxicity. In addition to the overexpression of HER1 in androgen- independent prostate cancer cells, the relative indolence of the disease will allow sufficient time for the immune system to develop meaningful antitumor responses.

Previously, preclinical studies using this strategy were performed in mice. In this case vaccination of animals with the ECD of autologous EGFR overcame the tolerance to self-EGFR, promoting highly specific IgG titers capable to inhibit EGFR+ tumor cell growth *in vitro*. This vaccination produced antimetastatic effect in 3LL-D122 Lewis lung carcinoma model (Ramirez et al., 2006, 2008) and did not have a deleterious effect on the healing process in adult mice (Fuentes et al., 2014). In addition, toxicological studies in rats and monkeys demonstrated low toxicity and very safe profile of the HER1 vaccine (Barro et al., 2012).

Since HER1 is widely expressed in normal human tissue, the first issue was to evaluate safety and tolerability of the vaccine. Theoretically, the treatment with the HER1 vaccine could produce a significant number of polyclonal antibodies that could be similar to approved monoclonal antibodies like cetuximab and panitumumab regarding fine specificity and avidity. It is known that both MAbs produce several toxic effects, such as, skin and mucous membranes lesions, digestive disorders, hypomagnesemia, among others (Bada et al., 2002). In addition, the induction of T cell response specific to HER1 might induce the killing of normal cells causing severe reactions such as organ-specific autoimmunity. This potential toxicity was the cause by which the initial dose of the vaccine was 100 μg. This dose was the half of the dose evaluated in monkeys (Barro et al., 2012; Mancebo et al., 2012), and it demonstrated antitumor effect in mice (Ramirez et al., 2006, 2008). Then, the dose was increased up to 800 μg without find any important toxic effect. In spite of the HER1 vaccine induced high titers of anti-HER1-ECD polyclonal antibodies and in some patients specific T cell response, the adverse events most frequently associated with the immunogen was pain at the administration site likely attributed to the oily adjuvant Montanide ISA 51 VG (Bada et al., 2005). Hence, we can conclude that the vaccine was safe and tolerable. Similar findings were observed in breast cancer patients treated with a recombinant HER2 protein. In this study, they found that frequency and severity of adverse events were similar across the different administered doses, showing that higher doses of HER2 did not lead to more severe adverse events (Limentani et al., 2016).

Regarding to the induced immune response, our study demonstrated that sequential vaccinations with HER1vaccine elicited both humoral and cellular response in patients. The vaccination generated high titers of polyclonal antibodies against the ECD of HER1. Antibodies were more quickly developed with the higher doses than with lower doses. Antibody production against EGFR family after vaccination has been already described, but specifically against the HER2 member. In previous study in which a HER2/neu vaccine adjuvanted in GM-CSF was administered to 35 patients with breast, ovarian or non-small cell lung cancer, 60% of patients developed IgG specific antibody response to at least one peptide included in their vaccine (Disis et al., 2004). Moreover in another study, an IgG specific antibody response was found in all women with metastatic breast cancer immunized with an HER2-based vaccine adjuvanted in AS15 and concomitantly treated with the tyrosine kinase inhibitor lapatinib (Hamilton et al., 2012). In this case the magnitude of specific antibody response (titers: 1:6400) was lower as compared with the anti-HER1 response obtained in our study. Our results evidenced that there was not a dose-dependency in the specific IgG antibody response, due to titers obtained were similar among all doses. Only a significant difference was seen with the lowest dose of 100 μg. In line with our findings, a phase I/II clinical trial in breast cancer patients treated with a recombinant HER2 protein adjuvanted in AS15 (Limentani et al., 2016) recently reported the best humoral response (higher frequency of responders and higher anti-HER2 geometric mean concentration) in the group received the highest dose of vaccine (500 μg) in comparison with the lowest one (20 μg). Furthermore, in our study a high antibody response was maintained at least 3 months after ending vaccination in most patients which could be related with the protein origin of the vaccine composition. It demonstrated that the vaccine can induce a memory immune response and would be interesting to determine how long this response last after stopping vaccination.

When subclasses analyses were performed, our results showed levels of IgG1 and IgG3 in the majority of patients, indicating a dominance of a Th1 response after HER1-ECD vaccination. This finding suggests the induction of CD8+ T cells recognizing different prostate cancer cells and multiple defined prostate cancer-specific epitopes (Wieckowski et al., 2011). This Th1-polarization in the humoral response is likely induced by the use of VSSP as adjuvant in the HER1 vaccine. Experiments with antigen-specific transgenic T cells demonstrated that VSSP-treated DCs induced a Th1 phenotype in stimulated naïve CD4+ T cells (Mesa et al., 2004). Furthermore, VSSP expanded CD8+ T cells specific for the co-injected antigen and promoted an effective *in vivo* cytotoxic T lymphocytes (CTL) response (Oliver et al., 2012). On the other hand, anti-HER1 monoclonal antibodies, which have been demonstrated good clinical results in cancer patients, are IgG1 antibodies (Dechant et al., 2008; Garrido et al., 2011; You and Chen, 2012) since IgG1 subclasses potently triggers effector mechanisms such as complement activation and antibody dependent cellular cytotoxicity (Garrido et al., 2011). Hence, the induction of IgG1 as predominant subclass should be crucial for the effectiveness of the vaccine. It is interesting to note that higher doses of vaccine induced the arising of IgG4 subclass. Indeed, two patients from 800 μg and one from 600 μg had IgG4 as predominant subclasses suggesting a Th2 pattern. The mechanisms by which these doses of vaccine drive the subclasses switching in some patients remain to be elucidated. Nevertheless, these findings should be considered for optimal vaccine doses in further clinical trials.

It was previously reported that patients vaccinated with 300 μg of a truncated 146HER2 protein complexed with nanogels of cholesteryl pullulan, developed anti-HER2 specific antibodies which failed to bind to HER2 on tumor cells surface (Kageyama et al., 2008). Similarly, our results demonstrated that sera from patients vaccinated with 100 and 200 μg of HER1 vaccine failed to recognize the tumor cell line A431 overexpressing HER1. However, antibodies induced with higher doses of HER1 vaccine (600 and 800 μg) recognize, although modestly, this receptor in its natural conformation in A431 cells membranes. In addition, patient’s hyperimmune sera, mainly from higher doses of the vaccine, were able to inhibit the EGF-induced HER1 phosphorylation as it was previously demonstrated in pre-clinical studies (Alpizar et al., 2009). In cancer patients, inhibition of HER signaling mediated by polyclonal antibodies have been also reported using a HER2-based vaccine in trastuzumab-refractory patients (Ren et al., 2012). In addition, it is strongly confirmed that MAbs specific to HER1 are able to inhibit the HER1 phosphorylation in tumor cell lines and, as consequence, tumor proliferation. Hence, the induction of polyclonal antibodies which recognize multiple epitopes on the ECD of HER1 is an appealing strategy for inhibiting EGFR signaling cascade. It could be more effective than a single targeting epitope by MAbs, issue that will be addressed in future studies (Pedersen et al., 2010).

Previous study published that hyperimmune sera obtained in non-small cell lung cancer patients treated with an EGF-based vaccine was able to inhibit the HER1 phosphorylation trough the anti-EGF specific antibodies. Notably, there was a significant direct correlation between the antibody titer and the abrogation of HER1 phosphorylation (Garcia et al., 2008). In contrast, we could not establish a correlation between the magnitude of antibody response and the functionality. Indeed, similar titers were induced with all the vaccine doses but 100 μg and only higher doses generated functional antibodies suggesting diverse qualities depending on the dose. Future experiments should address the characterization of the antibodies regarding to HER1 epitope recognition and affinities.

Although the importance of humoral immunity in anti-cancer immunosurveillance is being reconsidered, tumor-targeting humoral responses developing in a therapeutic setting are generally insufficient to mediate tumor rejection. Therefore, a therapeutic cancer vaccine must be capable of inducing a high number of antigen-specific T-cells against an established tumor, which can migrate to the tumor site and perform their effector functions (Nguyen et al., 2014). In this sense, HER1 vaccine was able to induce HER1-specific IFN-γ-producing T cells mostly in patients treated with higher doses of the vaccine (400, 600, and 800 μg) without an increase in toxicity. In line with our findings, in which higher vaccine doses seem to be crucial for induction of T cell response, a clinical trial was performed with E75, an immunogenic peptide derived from HER2/neu protein, in combination with GM-CSF (granulocyte-macrophage colony stimulating factor) as adjuvant. This trial was conducted on high-risk recurrence breast cancer patients demonstrated that clonal expansion of E75-specific CTLs peaked after 1 month of vaccination in a dose-dependent response manner (Peoples et al., 2005). It would be important to study whether specific T cells generated with the HER1 vaccine are able to infiltrate tumor lesions.

Notably, an association between immune response and OS was found in vaccinated patients. In spite of the small amount of patients, those with higher titers of anti-HER1 antibodies showed better survival times, although it was not significant. Few studies in the literature find association between humoral response and clinical outcome. In this sense, it was previously reported that patients immunized with an EGF-based vaccine showed better survival times in the group who elicited a good anti-EGF antibody response compared with the group who did not (Garcia et al., 2008). This finding was confirmed with larger patient sample in the recently finished phase III clinical trial (manuscript in preparation). Another exploratory study using a HER2 protein-based vaccine in HER2+ metastatic breast cancer patients found that a longer progression-free-survival time was observed for patients with high anti-HER2-ECD antibodies concentrations compared to low antibody response (Curigliano et al., 2016). In addition, in a phase III clinical trial in which Sipuleucel T was administered to CRPC patients, IgG response to PSAs was associated with improved OS in vaccinated patients (GuhaThakurta et al., 2015). These findings reconsider the humoral response as an important branch of the immune response taking into account the cytotoxic mechanism of actions of antibodies.

Regarding to cellular response, several studies in different tumor types, have reported an association with cellular immune response measured by IFN-γ-producing T cell ELISpot and improved clinical outcome (Disis et al., 1999). In the case of prostate cancer, a HER2 peptide-based vaccine demonstrated a trend in OS benefit for patients who developed positive IFN-γ-producing T cells during vaccination period (Peoples et al., 2005). In addition, in two phase II trials using PSA-TRICOM vaccine in mCRPC were found that the magnitude of tumor specific T cell response was associated with improved OS (Gulley et al., 2010; Kantoff et al., 2010).

These abovementioned studies including our, demonstrated that the development of high magnitude Type I immune response may be a predictor of clinical outcome. This preliminary result should be confirmed in future studies with larger number of patients.

In summary, our results suggest that HER1 vaccine is safe and immunogenic. Interesting, antibodies titers and T cell response seem to be associated with clinical outcome. Nevertheless, the complete mechanism of action of the vaccine and predictive biomarkers of clinical response remain to be elucidated. This study paves the way for optimally designing future larger clinical trials using this therapeutic cancer vaccine.

## Author Contributions

Conception and design of the study: IC, LA, XP, JR, JG, PL-L, BW, ES, TC, BS, AC, AM, and ZM. Clinical investigators (recruited and treated patients): IC, JR, and JG. Acquisition of data: IC, LA, AL, XP, MM, NG, KC, YuS, YaS, and MT. Analysis and interpretation of data: LA, AL, XP, MM, AC, BS, and ZM. Writing and/or revision of the manuscript: LA, TC, AC, and ZM. Final approval: LA, TC, AC, BS, and ZM.

## Conflict of Interest Statement

The authors declare that the research was conducted in the absence of any commercial or financial relationships that could be construed as a potential conflict of interest.

## Abbreviations

ECD: extra cellular domain
EGFR: epidermal growth factor receptor
HER: human epidermal growth factor
mCRPC: metastatic castration-resistant prostate cancer
OS: overall survival
PSA: prostate specific antigen
VSSP: very small size proteoliposome.
